# Decentralized Communication-Free Controller for Synchronous Solar-Powered Water Pumping with Emulated Neighbor Sensing

**DOI:** 10.3390/s25123811

**Published:** 2025-06-18

**Authors:** Roungsan Chaisricharoen, Wanus Srimaharaj, Punnarumol Temdee, Hamed Yahoui, Nina Bencheva

**Affiliations:** 1 Excellence Center in Industry 4.0, Computer and Communication Engineering for Capacity Building Research Center, School of Applied Digital Technology, Mae Fah Luang University, Chiang Rai 57100, Thailand; roungsan.cha@mfu.ac.th (R.C.); punnarumol@mfu.ac.th (P.T.); 2 Department of Information Technology, The International College, Payap University, Chiang Mai 50000, Thailand; wanus_s@payap.ac.th; 3 Département Mécanique, Université Claude Bernard Lyon 1, 69622 Lyon, France; hamed.yahoui@univ-lyon1.fr; 4 Department of Telecommunications, University of Ruse “Angel Kanchev”, 7017 Ruse, Bulgaria

**Keywords:** decentralized control, communication-free, synchronous control, solar actuation, water pumping, neighbor sensing

## Abstract

Solar-powered pumping systems using series pumps are commonly applied in the delivery of water to remote agricultural regions, particularly in hilly tropical terrain. The synchronization of these pumps typically depends on reliable communication; however, dense vegetation, elevation changes, and weather conditions often disrupt signals. To address these limitations, a fully decentralized, communication-free control system is proposed. Each pumping station operates independently while maintaining synchronized operation through emulated neighbor sensing. The system applies a discrete-time control algorithm with virtual sensing that estimates neighboring pump statuses. Each station consists of a solar photovoltaic (PV) array, variable-speed drive, variable inlet valve, reserve tank, and local control unit. The controller adjusts the valve positions and pump power based on real-time water level measurements and virtual neighbor sensing. The simulation results across four scenarios, including clear sky, cloudy conditions, temporary outage, and varied irradiance, demonstrated steady-state operation with no water overflow or shortage and a steady-state error less than 4% for 3 m^3^ transfer. The error decreased as the average power increased. The proposed method maintained system functionality under simulated power outage and variable irradiance, confirming its suitability for remote agricultural areas where communication infrastructure is limited.

## 1. Introduction

Water distribution systems in remote agricultural areas often rely on solar energy for pumping operations, necessitating multi-pump configurations to tackle long-distance delivery and significant elevation differences. Integrated solar water supply systems were evaluated in isolated agricultural areas in Thailand, demonstrating effectiveness in overcoming challenges associated with significant elevation differences through optimized pump configurations and tailored energy management strategies. We found that the systems achieved an average pumping efficiency of 85%, with energy consumption reduced by 30% compared to conventional systems. Additionally, the optimized configurations enabled water delivery over elevation differences exceeding 50 m, maintaining consistent flow rates of 2.5–3.0 L per second under variable solar conditions [1].

The geographical characteristics of such areas necessitate effective pump synchronization. Traditional methods relying on continuous communication between stations have proven unreliable, particularly in hilly tropical terrain where signal interference and power fluctuations frequently disrupt operations. Studies on distributed pumping systems in tropical conditions have reported signal disruption rates of up to 40% during peak solar radiation hours and power fluctuations exceeding 20% in unstable grid conditions, highlighting the need for alternative solutions [2].

Peer-to-peer energy distribution models have been explored as an alternative to centralized control in solar-powered pumping systems to address communication challenges. However, field measurements across various installations have identified significant limitations in low-voltage DC grid implementations, with transmission losses causing efficiency reductions of up to 25% at distances exceeding 500 m [3]. These findings emphasize the need for localized power generation at each pumping station, requiring the solar array capacity to be precisely matched to the specific demands of individual pipeline segments.

Stochastic processes, such as Markov chain prediction techniques, have been proposed to address the limitations of communication-dependent systems by estimating the flow conditions between adjacent stations during outages. Experimental data from field installations indicate that the methods achieve a prediction accuracy of 80–88% under stable conditions. However, the performance declines to approximately 65% during periods of rapid flow variation [4]. This reduction in accuracy is particularly problematic in hilly tropical terrains, where communication interruptions can be prolonged, emphasizing the need for more reliable flow control solutions.

Meanwhile, conventional valve-based flow control systems provide an alternative to interstation communication-dependent methods. Digital valve control implementations in irrigation systems have demonstrated a flow regulation accuracy of 93% without requiring data exchange between stations [5]. The integration of entry valves at each station allows for autonomous flow control based on the immediate upstream conditions, freeing the system from reliance on prediction algorithms and real-time data transmission. Additionally, valve-controlled systems operating with multi-pump installations maintain flow stability, whereas Markov chain-based systems exhibit a ±12% variation [6]. Furthermore, performance assessments under fluctuating solar conditions indicate that valve-controlled systems achieve 92% flow accuracy during power variations between 600 and 1000 W/m^2^, whereas communication-dependent systems demonstrate only 75% accuracy under the same conditions [7].

These studies provide valuable insights into solar-powered water distribution systems; however, they slightly overlook the key issues related to power instability that are inherent in solar energy applications. The variable nature of solar power, influenced by changing weather conditions and diurnal cycles, leads to difficulties in maintaining consistent pump operation and system efficiency. Addressing these issues is necessary for improving the reliability and performance of solar-powered water distribution systems, particularly in remote agricultural environments where power stability cannot be assured.

Dependence on complex communication infrastructure, prediction errors during flow variation, and efficiency loss under power instability remain unresolved in many control schemes. This study proposes a fully distributed, communication-free control system using digital valve control at each station. The proposed method allows each station to regulate its operation independently based on local sensor feedback, without relying on interstation communication or predictive synchronization.

Unlike conventional IoT-based SCADA systems and predictive control methods such as hidden Markov models or hybrid Bayesian networks, the proposed approach does not depend on real-time communication or model-based prediction. Instead, the system relies on local measurements and virtual outflow inference to manage pump operation. While prior studies have reported water savings using SCADA-based control, the performance under signal loss and rapid variation remains limited. The proposed method addresses this by avoiding communication and enabling station-level adaptation, which we have demonstrated across four simulation scenarios. This approach eliminates the sensitivity to signal degradation or connectivity constraints often encountered in LoRa or Sigfox-based systems.

## 2. Literature Review

The advancement of solar-powered water distribution systems is necessary due to the growing need for sustainable and energy-efficient agricultural irrigation solutions. Recent innovations in automation, control methodologies, and energy management strategies have improved system efficiency and operational reliability.

IoT-based supervisory control and data acquisition (SCADA) systems have been implemented to optimize irrigation control. An IoT-integrated SCADA system for banana crop irrigation demonstrated improved water management by enabling remote monitoring and automated distribution. This system facilitated real-time data acquisition, resulting in a 25% reduction in water consumption and a 30% improvement in irrigation scheduling accuracy [8]. However, the dependency on continuous communication infrastructure remains a challenge, particularly in remote agricultural regions where signal disruptions are frequent.

To address the communication constraints in decentralized water distribution, long-range communication technologies such as LoRa and Sigfox have been explored. These technologies provide low-power, long-range connectivity suitable for agricultural applications. Studies have indicated that LoRa networks can achieve communication distances of up to 15 km in rural areas with only a 5% data loss rate, making them viable options for remote irrigation monitoring [9]. Nevertheless, environmental conditions and terrain-related interferences can impact network stability.

Adaptive fuzzy proportional–integral–derivative (PID) controllers have been applied to improve water distribution system responses and stability. The implementation of adaptive fuzzy PID control for high-speed on–off valves in water hydraulic manipulators has shown a 40% improvement in response time and a 35% reduction in steady-state error compared to conventional PID controllers [10]. The effectiveness of PID control depends on precise system modeling and parameter tuning, which remain sensitive to variable solar inputs.

The efficient utilization of solar energy in water pumping has been addressed through maximum power point tracking (MPPT) techniques. A comparative study of MPPT methods identified perturb and observe (P&O) and incremental conductance as the most effective. P&O-based MPPT achieved up to 98% energy conversion efficiency under stable irradiance, while incremental conductance MPPT maintained better stability under fluctuating solar conditions [11]. The difficulty in sustaining pump performance during intermittent solar inputs remains a core limitation.

To mitigate failures in communication-dependent systems, data-driven models such as hybrid Bayesian networks have been adopted to predict flow conditions and detect faults. A simulation-based evaluation demonstrated that such models improve the predictive accuracy by 85% and increase fault detection rates by 90% [12]. However, the models’ performances decline during abrupt flow transitions, limiting their reliability under unstable operating conditions.

Reliable performance in solar-powered pumping also depends on fault detection and decentralized control. A review of diagnostic methods in energy systems highlighted that model-based and data-driven approaches can detect up to 95% of system anomalies. These results, derived from green hydrogen production systems, suggest an application potential in solar-powered water pumping [13]. Valve-based local control offers an alternative, removing dependence on communication or prediction and enabling resilience in signal-degraded settings.

Distributed control strategies specific to solar-powered water networks have also been developed. A decentralized scheme using a hidden Markov model improved communication efficiency in serial pump operations by predicting the state of remote units and adjusting system responses [14]. Additionally, a peer-to-peer (P2P) control framework for synchronous solar-powered serial pumps demonstrated consistent operation without battery storage, achieving a simulation measurement error of less than 4% under varying solar irradiance [15].

Optimization of photovoltaic-powered water pumping systems has been demonstrated through the combined use of Maximum Power Point Tracking using the Bat algorithm (MPPT-bat) and Direct Torque Control (DTC). The MPPT-bat technique improved the tracking speed and reduced oscillation under varying climate conditions by adapting power extraction from the photovoltaic generator. The DTC controller regulated the motor response to match available energy, minimizing torque-related inefficiencies and avoiding unsuitable load profiles. The coordinated MPPT-bat and DTC structure improved the overall system robustness, reduced tracking error, and enhanced the efficiency of pump rotation. These adjustments led to an increase in water delivery performance while maintaining energy stability during solar fluctuations [16].

A solar-powered water pumping system was developed using a Brushless DC (BLDC) motor driven by a photovoltaic array and regulated through an Artificial Neural Network (ANN)-based Maximum Power Point Tracking (MPPT) method. The configuration included a Zeta converter for voltage regulation, a Voltage Source Inverter for motor drive, and Hall sensors for commutation. The ANN-MPPT algorithm dynamically adjusted the converter output to maximize energy transfer under variable sunlight conditions. The system maintained a stable motor speed of 2500 rpm and delivered a peak power output of 410 W, demonstrating faster stabilization and lower power loss compared to traditional MPPT approaches. The design proved effective in off-grid agricultural areas with limited infrastructure, offering both environmental and performance benefits for irrigation [17].

Optimizing photovoltaic performance under non-uniform irradiance is critical in distributed water pumping systems. A hybrid MPPT control strategy combining perturb-and-observe and incremental conductance methods was proposed to address energy loss in partially shaded environments. The method was applied to Permanent Magnet Synchronous Motor (PMSM)-driven solar pumps, achieving improved tracking performance and enhanced pump efficiency under dynamic shading conditions [18]. This dual-mode approach contributed to achieving faster convergence and fewer oscillations around the power point, addressing one of the main causes of inconsistent flow delivery in terrain with intermittent shadows.

A detailed parametric analysis for converting conventional water supply systems to photovoltaic-driven operations was presented for small municipalities in Alicante. The study evaluated terrain elevation, solar resource variability, and system configuration requirements. The results highlighted that the storage capacity, inverter efficiency, and hydraulic lift were primary determinants of the transition feasibility. These findings underline the importance of site-specific modeling when designing a solar-based water supply for rural or semi-urban environments, complementing the decentralized strategies used in agriculture [19].

A transformerless single-phase photovoltaic pumping system was designed to operate a centrifugal water pump using a single-phase induction motor. The system architecture included a DC–DC boost converter with MPPT to extract maximum power from the PV array and a bidirectional buck-boost converter to manage battery charging and discharging between the load and the DC bus. A single-phase inverter converted the DC voltage into AC output, supplying the induction motor with a voltage/frequency (V/f) scalar-controlled input. Scalar motor control provided sufficient torque and speed to support the pump operation under varying PV and motor conditions. Simulation results confirmed efficient motor drive and adaptable water delivery, validating the configuration for remote irrigation use [20].

Claw-pole magnetic levitation torque motor (CPMLTM) was developed as an electromechanical converter for two-dimensional hydraulic valves. The CPMLTM architecture leverages cogging torque and axial recovery forces between the permanent magnet rotor and claw-pole stator, enabling automatic axial and circumferential neutral positioning. Structural optimization via finite-element response models allowed design enhancement of pole-tooth geometry and slot openings. Prototype testing demonstrated that current and displacement share a linear relationship within ±50 A, and the CPMLTM achieved 1.54× higher permanent-magnet utilization and a 33.6% mass reduction compared to moving-iron torque motors, significantly simplifying neutral adjustment and improving positioning accuracy [21].

Scenario-based stochastic Model Predictive Control (MPC) methods were reviewed for managing reservoirs, open channels, and urban water systems. These methods optimize over multiple plausible future inflow scenarios, based on historical data, to generate more robust control actions. The assessed studies demonstrate that such MPC frameworks improve resilience against hydrological uncertainties, more reliably meeting agricultural and urban supply demands, and outperform deterministic feedback-only schemes in handling extreme inflow variations [22].

SCADA-based irrigation automation improves water use efficiency under controlled connectivity. However, the monitoring reliability declines under poor communication conditions [8]. LoRa-enabled remote irrigation maintains connectivity over extended ranges but suffers from transmission degradation due to terrain variation [9]. Fuzzy PID controllers enhance transient stability in high-speed valve operations but require accurate system identification [10]. Hybrid Bayesian networks improve flow prediction accuracy under stable conditions but show a poorer performance during abrupt flow changes [12]. MPPT algorithms optimize solar power conversion but lack consistent flow maintenance under intermittent irradiance [11].

Previous implementations demonstrate specific limitations in terms of communication-dependent coordination, centralized processing, and sensitivity to fluctuating energy availability. Interestingly, a decoupled controller was introduced to treat a series of multivariable coupled systems for cascading pumping stations [23]. However, the controller was not fully dependent as feedback was needed from the neighboring station to satisfy its state variable. Therefore, valve-regulated, fully decentralized systems configured for local feedback control offer an alternative configuration under site-isolated and power-variable conditions.

## 3. Proposed Method

This study proposes a distributed control methodology for solar-powered agricultural pumping systems that operate independently of inter-station communication, while maintaining stable water flow despite the variability in solar PV power generation. The core objective is to design and implement a fully distributed, communication-free controller that controls the local pump and valve of a pumping station along a synchronous pumping line. The absence of any communication between neighboring stations is compensated for by an emulated sensor that virtually senses the behavior of the next station.

The control procedure begins by continuously monitoring three local parameters: the real-time solar power generation, the buffer tank water level *l*(*k*), and the outflow rate *Q*(*k*). If the water level falls below the lower threshold $L_{min}$, then the pump is immediately turned off to prevent dry running. The system then waits for sufficient inflow to restore the tank level before resuming operation. If the available solar power drops below the minimum operating level $P_{min}$, then the pump remains inactive and the system waits for improved irradiance.

Once the minimum water and power conditions are met, the system evaluates recent trends in pump power and outflow using a moving average approach. This allows each station to infer the readiness of its downstream neighbor using only local data, without any communication. If both the pump power and outflow increase, then the station assumes normal operation and slightly increases its output. If the pump power increases but the outflow decreases, this suggests that the downstream valve is closing, prompting a faster reduction in pump power using a scaling constant.

If zero outflow persists for a predefined delay interval, then a soft-start probing mechanism is initiated, gradually increasing the pump power in small steps to test the downstream readiness. If the downstream station still resists flow, then the probing sequence is aborted. In parallel, the inlet valve is regulated using a proportional controller based on tank level deviations. The valve closes when the level exceeds $L_{max}$, opens fully below $L_{min}$, and otherwise adjusts proportionally to maintain smooth regulation.

This autonomous and adaptive control logic loops continuously, enabling each pumping station to operate independently under varying hydraulic and environmental conditions. It also eliminates the need for synchronization signals or predictive communication-based coordination.

**Figure 1:**
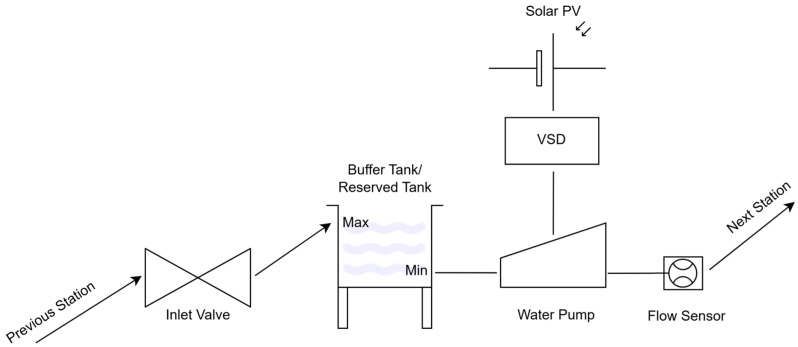
A pumping station and its components.

According to Figure 1, each station consists of a buffer/reserve tank or a direct water source, a solar PV system for power generation, a variable-speed drive (VSD) to power the pump, a variable inlet valve to regulate inflow, a flow sensor, and a pump to transfer water to the next station. The controller adjusts its inlet valve to regulate the flow into its tank and allocates power from its dedicated solar PV system to its pump, aiming to maximize water delivery while maintaining stable operation.

The system consists of multiple pumping stations arranged in series, each comprising a solar photovoltaic (PV) array, a variable-speed pump, an inlet valve, and a local control unit. Each station operates autonomously, regulating water flow based on the available solar power and real-time fluid-flow conditions. The system begins with the solar energy collection process, where the PV array at each station captures solar radiation and converts it into electrical power. The MPPT controller dynamically adjusts the voltage and current to maximize the power output based on the irradiance conditions for energy efficiency. This electrical power is then regulated and distributed to the system’s control unit, which is responsible for coordinating pump and valve operations. At the *i*th station, the variable-speed pump (*P_i_*) modulates its rotational speed to maintain a steady discharge flow (*Q_i_*), which is also affected by the valve of the next station (*V_i_*_+1_). The power allocated to the pump is influenced by the available solar power, the current water level within the reserved tank, and the valve position of the next station, which will be virtually sensed. Simultaneously, the inlet valve adjustment mechanism regulates the incoming water flow, retaining the water level within the predefined thresholds. The valve control unit operates based on a feedback loop that incorporates real-time water level measurements, dynamically adjusting the valve opening *V_i_* to prevent overflow and excessive fluctuations. The real-time feedback control mechanism continuously monitors the power availability, water levels, and flow rates. The control unit processes this information and adjusts the pump power $P_{i}$ and valve position *V_i_* accordingly to maintain steady-state conditions. It enables the smooth transition of water through the pumping stations without excessive pressure loss or cavitation.

Water transfer to the next station occurs as the processed flow is directed towards the downstream reservoir or the next pumping station. This cycle repeats sequentially until the final reserved tank is reached for an uninterrupted and self-regulating water distribution process. The decentralized control system operates independently of local solar power variations and hydraulic conditions. The controller adjusts its inlet valve to regulate the flow into its tank and allocates the power from its attached solar PV system to its pump, aiming to maximize water delivery for stable operation. The following sections describe the key control components and system settings.

### 3.1. Goal of the Control System

**Figure 2:**
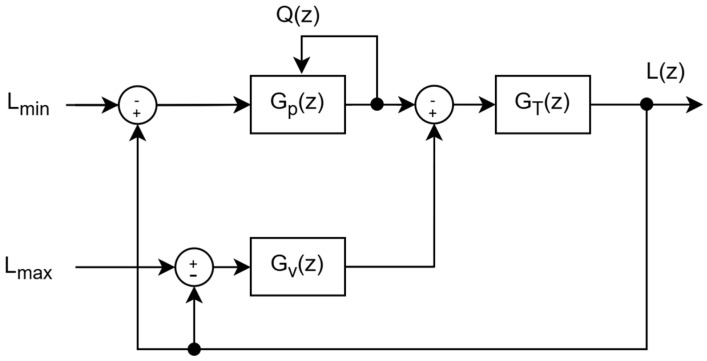
The control system.

The primary objective of the control system is to maintain the desired water level within a buffer/reserve tank. This is accomplished through the integration of two separate but goal-sharing controllers: one dedicated to the inlet valve and the other to the pump. The controllers collaboratively operate to maintain the water level consistently within the predefined minimum and maximum thresholds, as shown in Figure 2. The control system manages water level retention within predefined thresholds by dynamically adjusting the inlet valve and pump power. The system regulates the current water level *L*(*z*) based on the interaction between controllers, the tank dynamics, and the outflow sensed (*Q*(*z*)). *G_T_*(*z*) represents the tank dynamics, modeling how the net inflow affects the water level. The inlet valve controller *G_V_*(*z*) adjusts the valve operation, influencing the inlet flow, while the pump controller *G_P_*(*z*) regulates the pump operation, controlling the outflow *Q*(*z*), which is fed back as another input of *G_P_*(*z*).

To maintain stability, the system continuously monitors *L*(*z*) within the predefined limits, the minimum allowable level *L_min_*, and the maximum allowable level *L_max_*. The thresholds are reference constraints, guiding the control actions of both the inlet valve and the pump to prevent the overflow or depletion of the tank. The feedback loops continuously adjust in real time to correct deviations and maintain *L*(*z*) within the desired range. Each station maintains suitable pump power for sustained operation by continuously monitoring the outflow, which is also influenced by the valve of the next station (*V_i_*_+1_). The regulation of tank levels is achieved through adaptive valve control and pump modulation, allowing for smooth and efficient water distribution. This approach prevents overflow, dry running, and excessive pressure variations to achieve reliable long-term performance in remote agricultural applications.

### 3.2. Inlet Valve Controller

The inlet valve controller operates as a discrete-time proportional controller. This control methodology was chosen for its simplicity and effectiveness in responding to dynamic changes in the water level. The proportional control mechanism adjusts the valve opening $v\left(k\right)$ proportionally to the percentage of difference between the maximum water level (*L_max_*) and the current water level (*l*(*k*)), as shown in Equation (1):

(1) $$v\left(k\right)=\left\{\begin{array}{c} V_{max}K_{p}\left(\frac{L_{max}-l\left(k\right)}{L_{max}}\right),\;if\;{0\;\leq V}_{max}K_{p}\left(\frac{L_{max}-l\left(k\right)}{L_{max}}\right)\leq \;V_{max}\\ 0,\;if\;V_{max}K_{p}\left(\frac{L_{max}-l\left(k\right)}{L_{max}}\right)<0\\ V_{max},\;if\;V_{max}K_{p}\left(\frac{L_{max}-l\left(k\right)}{L_{max}}\right)>V_{max} \end{array}\right.$$

where *K_p_* is the proportional gain and $V_{max}\;$ is the maximum opening of the valve. The output valve position v(k) will be within the following range:

(2) $$0\;\leq v\left(k\right)\leq \;V_{max}$$

The choice of *K_p_* will determine how long the valve remains fully open and how fast it will be closed. The simplicity of monitoring the water level significantly reduces the likelihood of instability, as it prevents the system from overreacting to the transient fluctuations of the inflow, which can be of higher order as multiple factors are being counted. The valve control maintains the desired fluid level in a tank in order to avoid exceeding its physical limits while maintaining efficient level regulation.

### 3.3. Pump Controller with Emulated Neighbor Sensor

The pump controller is designed to regulate water outflow based on the generated solar power, the water level in the reserve tank, and the condition of the next station, approximated using outflow analysis. The outflow analysis can be viewed as an emulated neighbor sensor that will suggest the neighbor’s situation to the pump controller. The key to this approximation is to observe the moving average of the pump power and outflow to enhance stability and minimize fluctuations. Given that *k* is the current sequence, the *i*-sequence delayed moving average power is formulated as in Equation (3).

(3) $$P_{a}[k-i]=\frac{1}{N}\sum_{j=0}^{N-1}P[k-\left(i+j\right)]$$

where *N* represents the averaging window. Likewise, the *i*-sequence delayed moving average outflow is shown in Equation (4).

(4) $$q_{a}[k-i]=\frac{1}{N}\sum_{j=0}^{N-1}q[k-\left(i+j\right)]$$

The averaging techniques help smooth out variations in power and flow, leading to more stable control responses. The purpose of analyzing these delayed moving averages is to determine the relationship between the input pump’s power and the corresponding outflow. The key is to observe a change in the outflow as the response to a change in the input power. Based on the general fluid mechanic that requires a certain interval before allowing an input power to have an effect on the outflow, $q_{a}[k]$ is considered to be the response of $P_{a}[k-1]$, and $q_{a}[k-1]$ is considered to be the response of $P_{a}[k-2]$. These four delayed moving averages are key to the emulated sensing mechanism toward the next neighbor. The pump controller can better anticipate changes in demand and adjust its operation accordingly by incorporating this emulated sensor. The entire operation is started by determining the estimated power of the current sequence k ($p_{e}\left(k\right)$) through the following hierarchical piecewise function, shown as Equation (5):

(5) pek=pk−1,if qak=qak−1pk−1+∆p, if qak>qak−1pk−1−ks∆p, if qak<qak−1,if pak−1≥pak−2pk−1, if pak−1<pak−2,if qak−1>0 and pak−2>0 0, if k<Dpk−1+∆p, if k ≥D,if qak−1=0 or pak−2=0

The first part, which meets the condition $“q_{a}\left[k-1\right]>0\;and\;p_{a}\left[k-2\right]>0$”, describes a normal operation in which the outflow and the pump have not previously stopped. Under this condition, the values of $p_{a}\left[k-1\right]$ and $p_{a}\left[k-2\right]$ are compared. In the case that $p_{a}\left[k-1\right]\geq p_{a}\left[k-2\right]$, the recent average power is increased, which helps determine $p_{e}\left(k\right)$ based on the compared amount of $q_{a}\left[k\right]\;$ and $q_{a}\left[k-1\right]$. Normally, if the input power has increased and the responded outflow has also increased, then the current $p_{e}\left(k\right)$ can be increased by a small amount ${∆}_{p}$. However, if the valve of the next station (*v_i_*_+1_) is closing, then a decrease in outflow can be observed while the input power is increased. Therefore, $p_{e}\left(k\right)$ must be decreased by ${k_{s}∆}_{p}$ where $k_{s}$ is the stepping constant, to accelerate the decreasing sequence. This represents the fundamental logic behind the emulated sensing mechanism, allowing it to determine the condition of the next station without any communication as the outflow itself indirectly states the circumstance. The process of decreasing power is accelerated by $k_{s}$ because there is only one condition to decrease the input power compared to the conditions to maintain or increase it, which implies that the situation is dire and in need of a fast response.

The second part is when the outflow or the pump has stopped, as in the condition “$q_{a}\left[k-1\right]=0\;or\;p_{a}\left[k-2\right]=0$”. In this case, the delay sequence (*D*) is implemented to let $p_{e}\left(k\right)$ be zero until an intended delay interval has passed. In detail, *D* is based on the sampling time *T_s_* and a predefined delay time *T_D_*, as shown in Equation (6):

(6) $$D=\left\{\begin{array}{c} k+\left[\frac{T_{D}}{T_{s}}\right],\;if\;q_{a}\left[k-1\right]=0\;or\;P_{a}\left[k-2\right]=0\\ 0,\;otherwise\; \end{array}\right.$$

After the delay, a soft start will be initiated by gradually increasing the input power by ${∆}_{p}$ in the following sequences, which will eventually lead the system back to normal operation if there is no valve block at the next station. This mechanism also periodically probes the situation at the next station. If the next station is still not ready to receive inflow, then its valve will remain closed, which can be virtually sensed through the decreasing outflow. Thus, the power-decreasing mechanism in the first part will be activated and gradually shut down the probing operation. The real current power *p*[*k*] will be determined based on the conditionally calculated $p_{e}\left(k\right)$, the current generated power $p_{g}\left(k\right)$, and the current water level in the reserve tank *l*(*k*), as shown in Equation (7):

(7) $$p\left[k\right]=\left\{\begin{array}{c} \left\{\begin{array}{c} p_{e}\left[k\right],\;if\;0\leq \;p_{e}\left[k\right]\;\leq \;p_{g}\left(k\right)\\ 0,\;if\;p_{e}\left[k\right]<0\\ p_{g}\left(k\right),\;if\;p_{e}\left[k\right]>p_{g}\left(k\right) \end{array}\right\},\;if\;P_{i}[k]\geq P_{min}\;and\;l\left(k\right)>L_{min}\;\\ 0,\;otherwise \end{array}\right.$$

This final step serves to guarantee that the pump will have enough power to operate when the generated power is greater than or equal to the minimum required power *P_min_*. In addition, it also ensures that the reserve tank will not be emptied when the pump operates if and only if the current water level $l\left(k\right)$ is greater than the minimum level $L_{min}$. In summary, the algorithm dynamically adjusts the effective power output, using an adaptive proportional gain. This control strategy eliminates reliance on predictive algorithms and communication-dependent synchronization.

**Figure 3:**
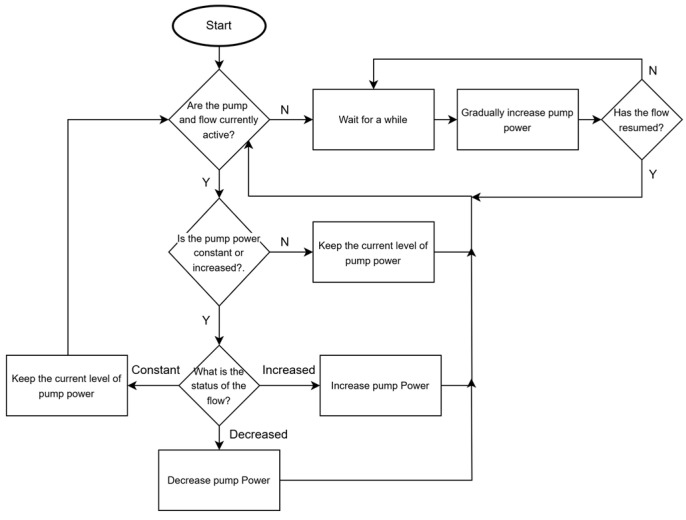
The pump control system.

Therefore, the proposed logic is important in maintaining functionality in remote installations where rapid environmental changes or unexpected disruptions frequently occur. The integration of a decentralized architecture allows each pumping station to perform operations autonomously and eliminates prior dependence on predictive synchronization or communication infrastructure. The combination of direct measurement, localized control, and adaptive flow regulation enhances system resilience and stable operation under varying solar conditions. Figure 3 presents a flowchart that simplifies the process of pump control for better comprehension.

The decision process begins by checking whether both the pump power (P) and flow (Q) have stopped. If both are inactive, then the system initiates a delay before proceeding. After the delay, the controller tries to soft-start the pump by gradually increasing its input power. Meanwhile, the flow is continuously measured, and if there are signs of increasing flow, then the pump will continue to the main logic loop of checking the flow and adjusting the pump power. In addition, almost all decisions are based on differential sensed data, which means that the system is more robust to small sensor deviation as long as the sensor’s sensitivity is at a certain level. For example, if we consider a reading from a flow sensor with a small error as q[k]+∆ and its next reading as $q\left[k+1\right]+∆$, then, when assessing the difference in making a decision, a small sensor deviation can be ignored, as they will cancel each other out through differencing. Hardware or component failure management is also partially implemented in the system, as any misalignment between power and flow will cause the system to eventually stop.

## 4. Simulation Results

The proposed system was implemented and evaluated in a high-fidelity simulation environment that replicates the real-world physical constraints of hydraulic flow, solar energy availability, and pump operation based on the physical setting described in the previous work [15], which provides a rather complex scenario involving unbalanced pumping loads between sections. The implementation is based on MathWorks’ Simscape, a simulation platform well regarded for its practical accuracy, often producing results that closely match experimental data in multi-domain system simulations [24,25,26].

### 4.1. System Configuration and Technical Specifications

**Figure 4:**
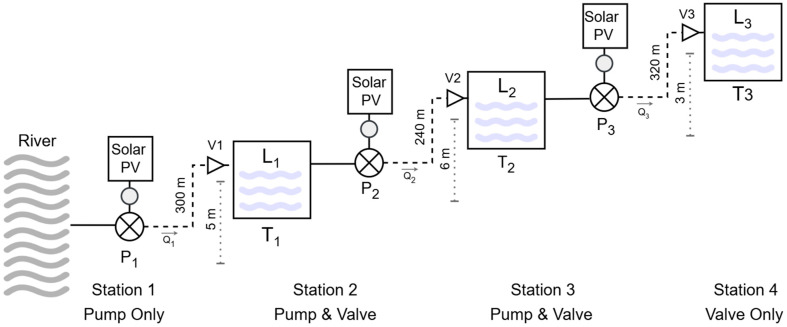
Simulation scenario.

According to Figure 4, the setting has four stations with different sets of devices. The first station is directly connected to a small river, which provides a virtually unlimited source of water. Therefore, this station does not require a valve to control the input flow. The second and third stations are intermediate stations and require all the components shown in Figure 2. The fourth station, which controls the flow into a final reserve tank, requires only an inlet valve and its controller.

All stations are coupled with a 2-inch pipe; these are of various lengths and heights. The components of all stations are described in the following Table 1.

Each pump is independently powered by its own solar PV array, improving reliability and enabling modular expansion. Water is lifted in stages, minimizing energy losses and allowing adaptation to terrain elevation changes. The decentralized approach facilitates future expansion, as additional stages or capacity can be added with minimal interference to the existing system.

The buffer tanks at the intermediate stations have a capacity of 1 cubic meter and a height of 1 m, meaning that the water level is equal to the water volume. The reserve tank at the final station has a capacity of 10 cubic meters based on a 2 m height. The specifications of all tanks and the corresponding control parameters, as defined in Equations (1)–(7), are listed in as follows in Table 2. Although this setting only involves four stations, it is one of a series of multivariable coupled systems, which are considered complicated and difficult due to the nature of the coupling relationship [23].

At Ruse University, the water dynamics were modeled using MathWorks’ Simscape, which provides reasonable physical constraints. The pumps in the model are 3 hp centrifugal pumps with standard parameters, which are common in agricultural irrigation systems. The inlet valves were modeled as modulating valves based on a standard 2-inch model. The pump of each station is powered by a VSD based on the generated power of a 3 hp Solar PV system. All pumps are simulated with 70% efficiency, which is common in agricultural practice. The simulation consists of four scenarios with different power parameters, based on regular practice in hilly tropical regions that typically have three seasons: summer, rainy, and winter. The first scenario is based on clear sky conditions, where the Solar PV receives most of the irradiation, as in the summer or winter seasons. The second scenario simulates a cloudy sky, emulating over half of the days during the rainy season. The third scenario is based on a clear sky, but with a temporary power outage at an intermediate station. The final scenario involves varied average power to demonstrate the reliability and flexibility of the proposed system. All scenarios are summarized in Table 3.

### 4.2. Clear Sky Conditions

**Figure 5:**
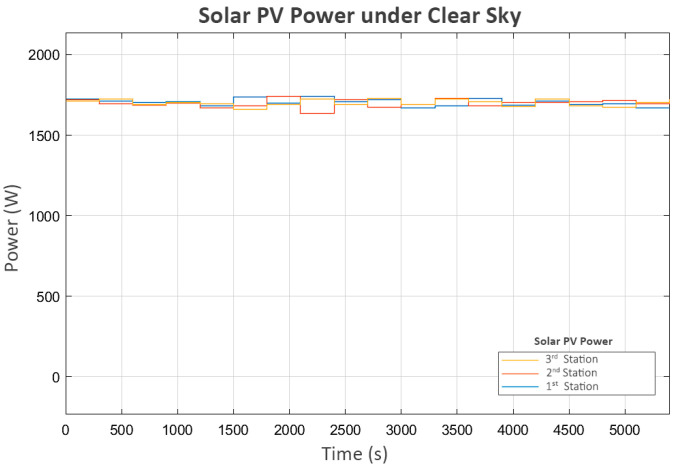
Solar PV power under clear sky.

The first scenario evaluates the system under clear sky conditions, where each station generates an average of 1700 Watts of solar power, as shown in Figure 5. A small variation in power is included to emulate the regular conditions of solar PVs in hilly terrain.

**Figure 6:**
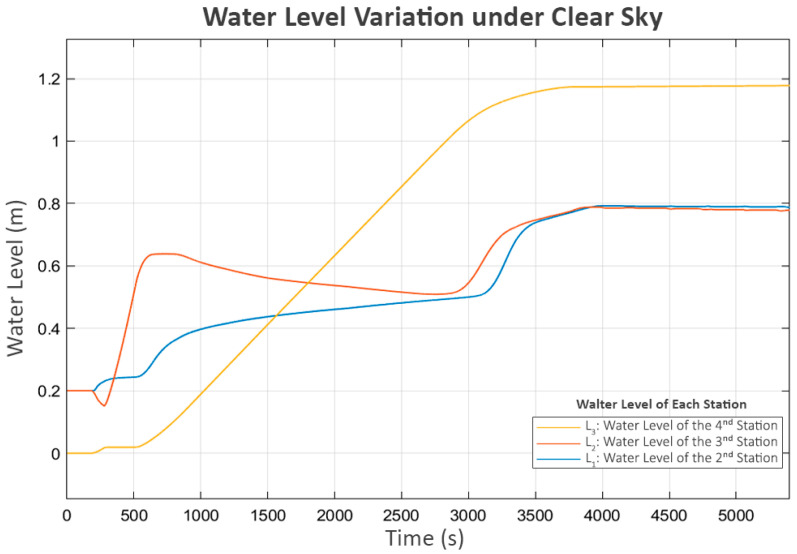
Water level variation under clear sky.

The first parameter under analysis is the level of the buffer and reserve tanks, shown in Figure 6, as it affects and is affected by all other parameters. L_1_ represents the level of the buffer tank at the second station, L_2_ represents the level of the buffer tank at the third station, and L_3_ represents the level of the reserve tank. L_3_ increases cumulatively, while L_1_ and L_2_ vary within the minimum and maximum levels.

**Figure 7:**
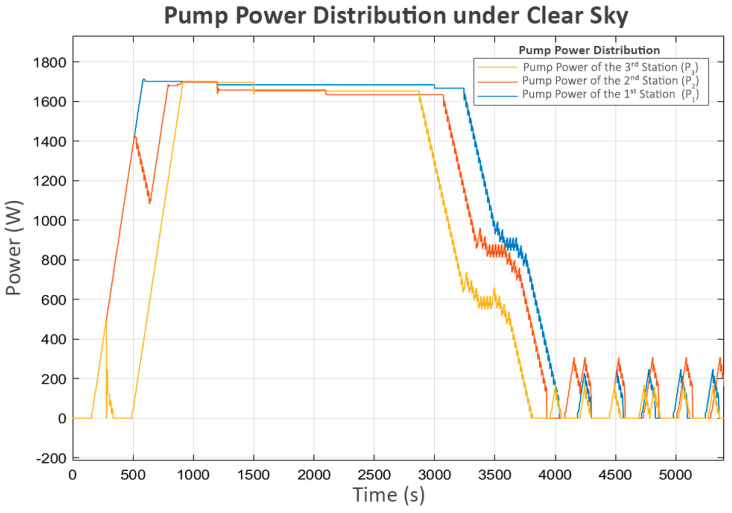
Pump power distribution under clear sky.

There is a short period of stalling in L_3_ during the initial runtime, which aligns with the brief off period between the delivered power and the pump at the third station (P_3_), as shown in Figure 7. This occurs because, during this period, the water level in its buffer tank (L_2_) is low and close to the minimum level. As a result, its pump (P_3_) temporarily stops. This short shutdown of P_3_ allows L_1_ and L_2_ to recover, enabling the subsequent continuous operation of all stations.

**Figure 8:**
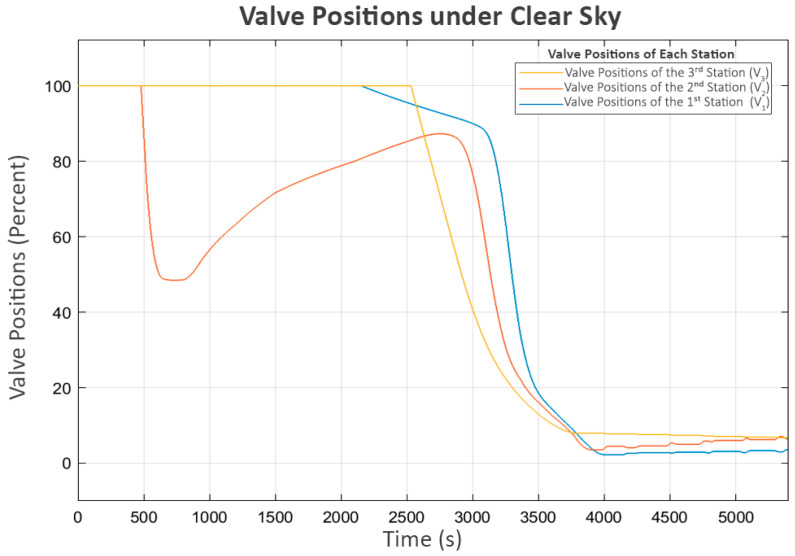
Valve positions under clear sky.

During the period when P_3_ briefly stopped, the sharp rise in L_2_ reached its maximum boundary, causing its inlet valve (V_2_) to close rapidly, as shown in Figure 8. This situation led to a brief decrease in the power of the second pump (P_2_), which delivers flow from L_1_ to L_2_. This indicates that the system’s response was consistent with the control behavior expected of the proposed neighbor-sensing mechanism, as the second station, which controlled P_2_, could detect the closing of the valve at the third station (V_2_) and correctly reduce the power to P_2_.

**Figure 9:**
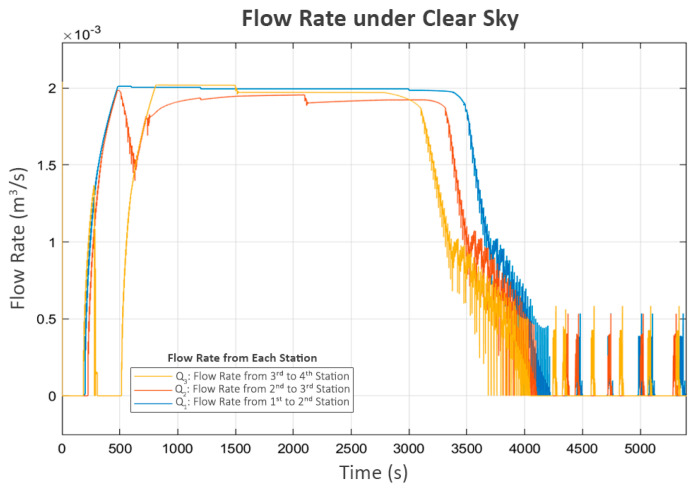
Flow rate under clear sky.

After a brief period of turbulence during the initial operation, the system resumed its expected behavior, as shown in the water levels in Figure 6, which correspond with the flow patterns in following Figure 9. The observed flows align well with the associated pump power levels. However, in the final phase, as L_3_ approached its limit, the inlet valve of the fourth station (V_3_) rapidly closed, significantly reducing its inflow (Q_3_), as shown in Figure 9. This event triggered the third station to reduce its pump power (P_3_), which led to an increase in its buffer tank level (L_2_) since its outflow decreased while the inflow remained approximately the same. As a cascading effect, the inlet valve to L_2_ (V_2_) was closed to keep the water level below the maximum threshold. Subsequently, the second station detected the changes at the third station and responded similarly, reducing the power of its pump (P_2_) and increasing its water level (L_1_), ultimately leading to the closure of its inlet valve (V_1_).

Finally, the first station recognizes the closure of V_1_ at the second station due to the decrease in its flow (Q_1_). Consequently, it turns off its pump (P_1_). However, as the control system is designed to be adaptive, a soft-start mechanism that increases the power to the pump in small increments is periodically attempted after a certain delay, as shown in Figure 7 and Figure 8. This mechanism allows each station to assess whether the next station is ready to accept flow while preventing damage to its own pump. Since the system has already reached a steady state and no further flow is needed, Figure 6 and Figure 9 show that these trial activations turn off after a short period. This is because the flow sensed during these trials consisted of unstable turbulence from pump startup rather than a stable, sustained flow. As a result, these trials were quickly terminated.

### 4.3. Cloudy Sky Conditions

**Figure 10:**
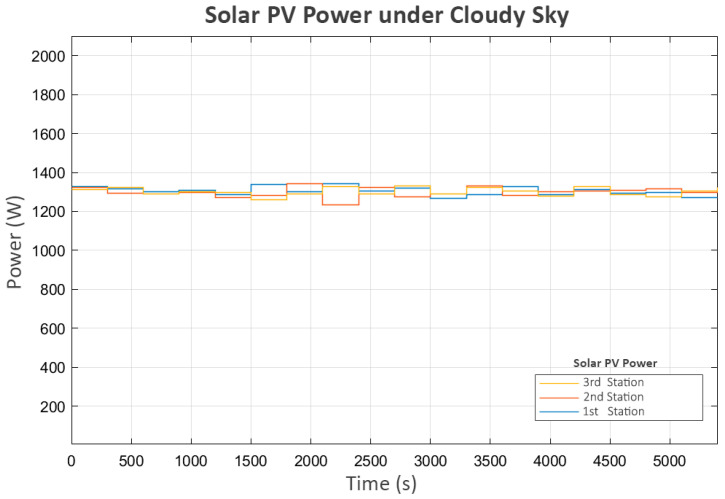
Solar pv power under cloudy sky.

**Figure 11:**
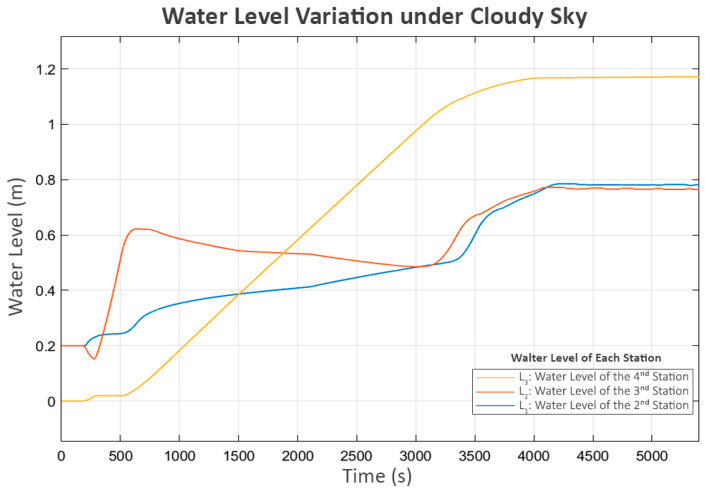
Water level variation under cloudy sky.

In this scenario, each station generated approximately 1300 Watts, with slight variations, as shown in Figure 10:

The water levels of all tanks are shown in Figure 11. The behavior closely resembles that observed under clear sky conditions, albeit with significantly slower dynamics. L_3_ reaches its boundary at around 4000 s, compared to 3500 s in the clear sky scenario.

**Figure 12:**
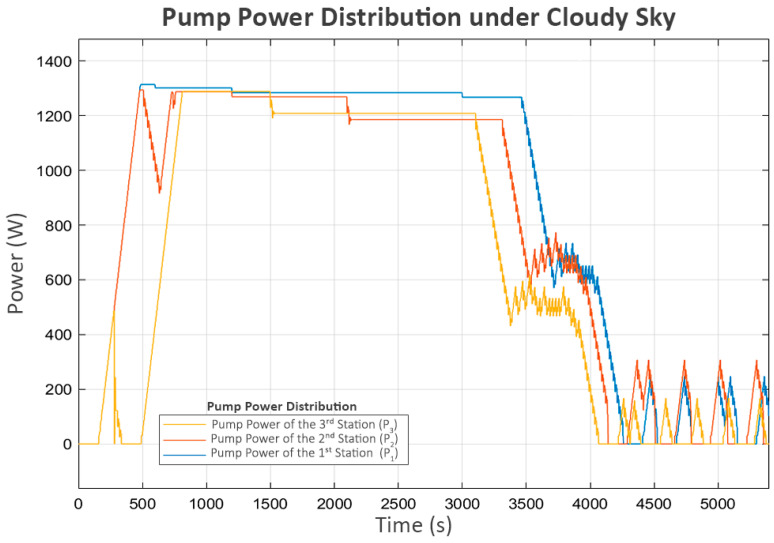
Pump power distribution under cloudy sky.

**Figure 13:**
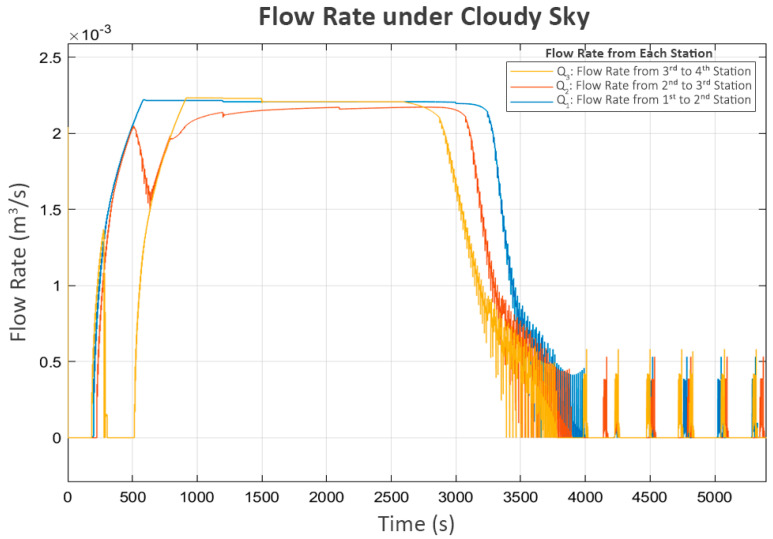
Flow rate under cloudy sky.

The power and flow for this scenario, shown in Figure 12 and Figure 13, followed similar patterns in the clear sky condition. The only difference is the slower convergence speed due to the lower power generated.

Trial turn-ons are also practiced in this scenario after the system approaches a steady state. The turbulence in flow, as shown in Figure 13, directly corresponds with the trial power supply activations in Figure 12. The short on–off pattern remains the same as in the clear sky scenario.

**Figure 14:**
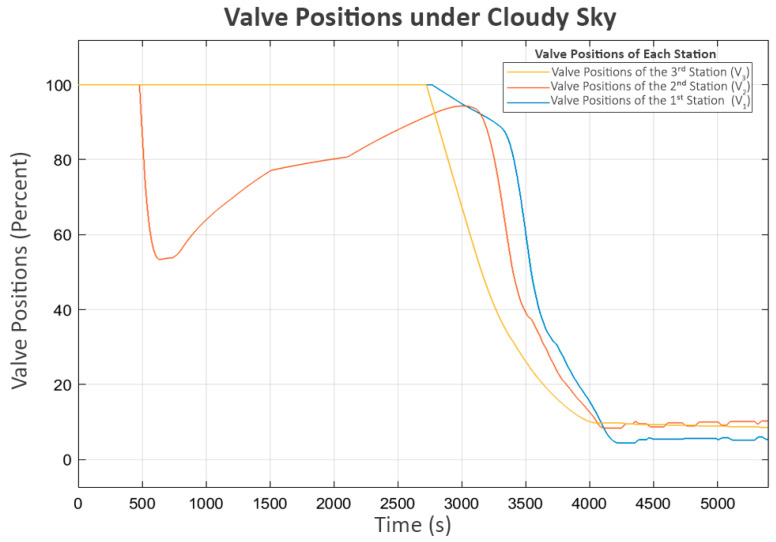
Valve positions under cloudy sky.

Accordingly, as shown in Figure 14, all valves follow the water levels in the same manner as in the first scenario.

### 4.4. Clear Sky with Temporary Power Outage

**Figure 15:**
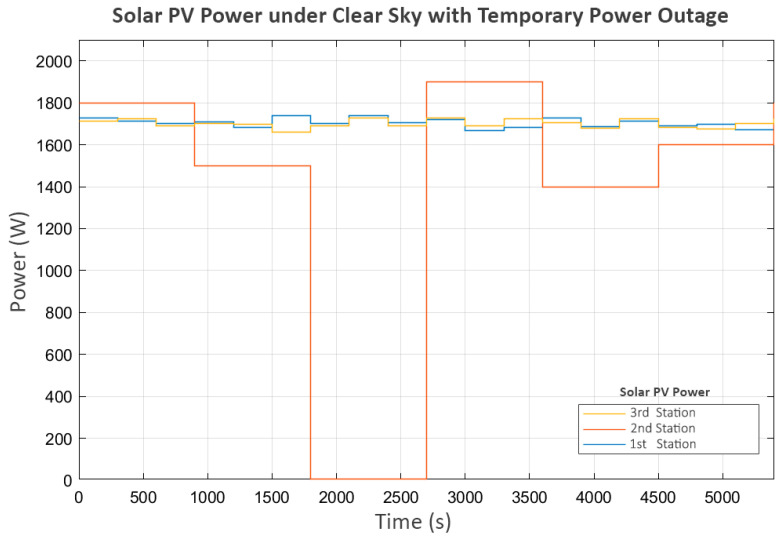
Solar PV power under clear sky with temporary power outage.

This scenario is based on the first and third stations each receiving an average of 1700 Watts as the clear sky scenario. The difference lies in the second station, which experiences a 15 min power outage and must suddenly shut down its pump. The power distribution for this scenario is shown in Figure 15.

**Figure 16:**
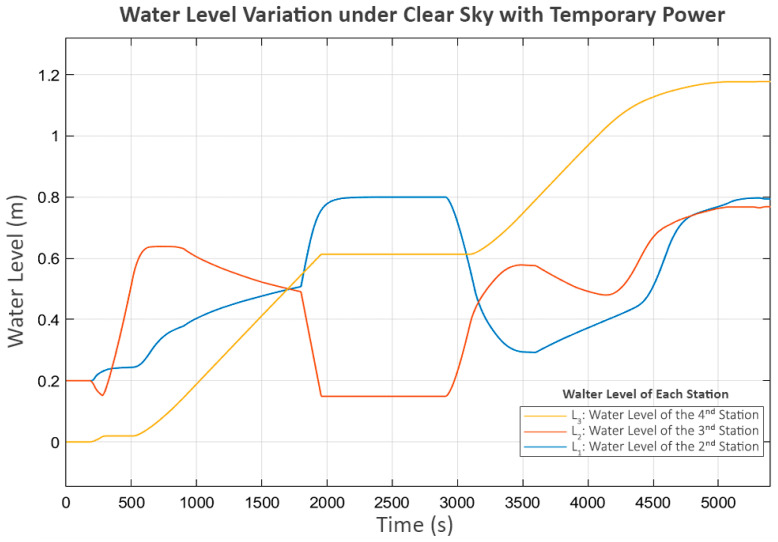
Water level variation under clear sky with temporary power outage.

According to Figure 16, the water level initially exhibits a 15 min stagnation at L_3_, beginning around the 2000th second and lasting until the 3000th second.

**Figure 17:**
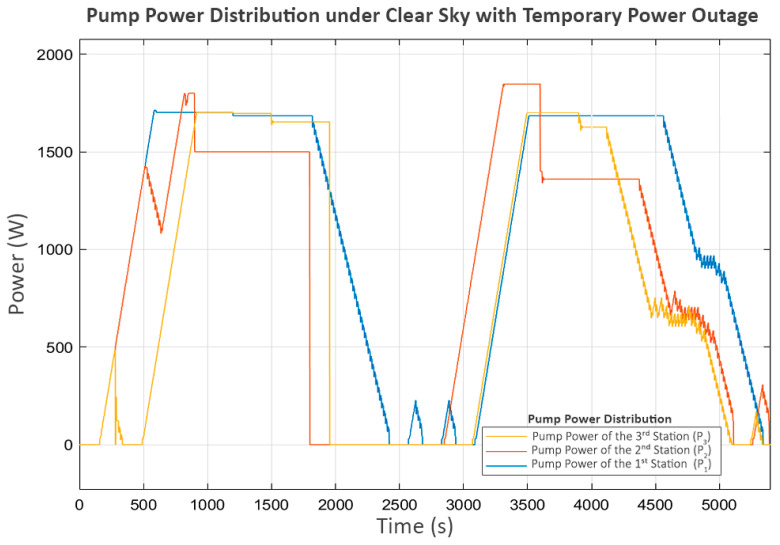
Pump power distribution under a clear sky with temporary power outage.

However, the power outage at the second station begins around the 1800th second. This suggests a significant delay, shown in the power graph in Figure 17, where P_2_ suddenly stops as soon as the power is cut, while P_3_ continues operating until the water level in its buffer tank (L_2_) reaches its minimum level.

**Figure 18:**
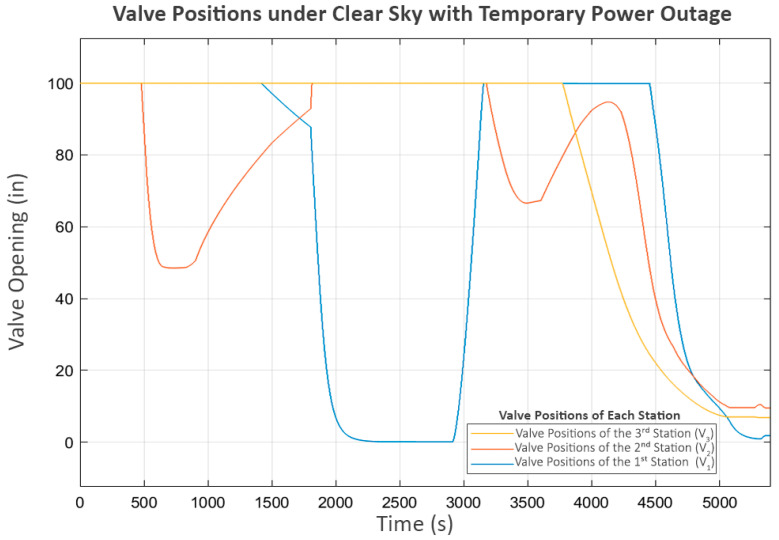
Valve positions under clear sky with temporary power outage.

At this point, P_2_ and P_3_ were completely stopped, but P_1_ at the first station was still operating. As a result, the water level at the second station (L_1_) increased until it reached its limit, eventually causing its inlet valve (V_1_) to close, as shown in Figure 18.

**Figure 19:**
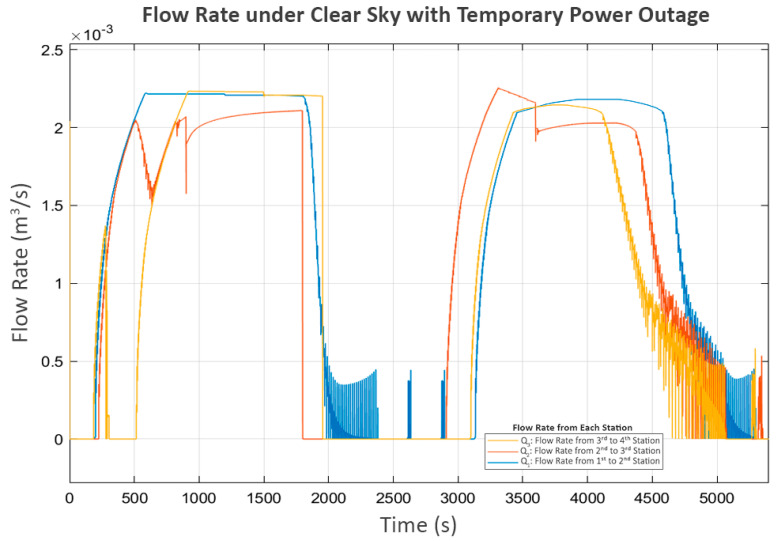
Flow rate under a clear sky with temporary power outage.

This action affected the flow from the first to the second station (Q_1_), shown in Figure 19, where the first station detected that the second station could no longer receive inflow. Consequently, it decreased the power of P_1_ until it reached a shutdown state, as shown in Figure 17.

At this stage, P_2_ shuts down due to a power outage, while P_3_ shuts down because of a lack of water. As a result, these two pumps do not perform the trial soft starts, as the status prohibits any pump operation according to Equation (7). However, P_1_, which still meets all the minimum requirements of Equation (7), continues to exhibit trial soft starts. The first two attempts fail, but the third attempt succeeds, because, at that point, power at the second station is restored, allowing P_2_ and P_3_ to resume operation.

In the later part of the process, all pumps continue operating until L_3_ reaches its maximum level, leading to a progressive shutdown in upstream components, reflecting inter-station regulation. In the final stage, when all tanks have reached steady-state levels, the power and flow graphs indicate that trial soft starts occur in P_2_ and P_3_. This scenario demonstrates the system’s performance in handling severe disturbances, ensuring that normal operation can be resumed despite significant disruptions.

### 4.5. Performance Under Varied Average Power

In this test, the performance of the proposed system is evaluated based on the time required to deliver 3 cubic meters of water under varying average power levels at each station. The minimum average power is set at 1000 Watts, increasing by 250 Watts in each step until reaching 2000 Watts, as shown in Table 4. The results indicate that the system performance correlates directly with the available solar power.

The final test evaluates the system’s performance across different average power levels, ranging from 1000 to 2000 Watts per station, increasing in 250-Watt increments. For each case, the simulation was executed until a total of 3 cubic meters of water had been transferred to the final reserve tank. The system consistently completed the task without any overflow or shortage across all power levels.

In addition to the filling time, steady-state power errors were analyzed during the final 300 s of each simulation after reaching operational stability. The errors were calculated as the absolute deviation between the commanded and actual pump power values. Table 4 shows that the system maintained steady-state power control within a maximum error of 3.4%, decreasing with increased available power. These results indicate that the control method ensures both flow reliability and efficient energy usage as the solar power availability changes.

## 5. Discussion and Conclusions

This study introduces a fully decentralized, communication-free control strategy for solar-powered pumping systems, incorporating emulated neighbor sensing and adaptive control to regulate flow in the absence of inter-station communication. A simulation across four scenarios, including clear sky, cloudy, power outage, and variable irradiance, showed that the system maintained regulated flow to avoid overflow or shortage events and respond appropriately to changes in power input.

Compared to communication-based systems, the proposed method eliminates the risk of performance degradation due to signal loss or network failure. While existing SCADA and LoRa systems rely on consistent connectivity, our solution achieves autonomous regulation using only local measurements and virtual sensing, lowering infrastructure and maintenance costs. The elimination of communication hardware and maintenance overheads in the proposed system may further reduce both capital and operational costs. Future work will integrate a detailed techno-economic model to evaluate cost-effectiveness relative to centralized or communication-reliant solutions. Table 5 summarizes the operational characteristics observed during simulations. Across all test conditions, the system avoided overflow and shortage, while adjusting pump and valve operation in response to power variability without requiring communication between stations. Moreover, the decentralized approach demonstrated recovery capability after a temporary shutdown, with flow and power dynamics stabilizing automatically upon the restoration of power availability.

Although the control logic includes adaptive features such as soft-start mechanisms and probing trials, the current framework does not yet fully account for component failure or sensor inaccuracies. These aspects are critical in long-term field deployment. As a next step, fault injection simulation and sensor noise modeling will be incorporated to evaluate the system’s tolerance to actuator failure, flow sensor drift, and signal degradation. The refinements are necessary to develop a complete fault-resilient deployment model.

Additionally, system scalability is a key area of concern. While the current four-station configuration is effective, further study is needed to assess how the system performs with more nodes and whether control stability can be maintained with increased hydraulic delay or varied elevation profiles. However, this problem is based on the needs of non-enterprise farmers, who generally prefer a robust system that does not require complex installation and maintenance. Therefore, systems for longer distances that require more than four stations are not as commonly required by the project’s target users.

From an economic standpoint, the proposed system offers reduced installation and operational costs by eliminating communication modules (e.g., LoRa gateways or cellular modems) and technician requirements. A preliminary cost comparison shows potential savings of up to 25–30% compared to communication-based counterparts and maintenance, particularly when deployed across long distances with no signal infrastructure.

In summary, the proposed system was designed for ease of installation, simple maintenance, and the prevention of communication issues, but it can suffer from longer converging times or slower responses, representing a trade-off against its simplicity. It represents a direct solution to a major issue faced by non-enterprise farmers, especially in developing countries, where complicated high-efficiency systems can be inconvenient, as a lack of resources and technical workforce is very common.

## Figures and Tables

**Table 1:** Specifications of the simulation scenario.

Station	Components	Specifications	Description
Station 1	Pump (P_1_) Solar PV panel Flow rate indicator (Q_1_)	2-inch-diameter pipe Length: 300 m Height difference: 5 m	Initial pumping station directly connected to river water source No valve required as river provides unlimited water supply
Station 2	Pump (P_2_) Valve (V1) Buffer tank (T_1_) with level indicator (L_1_) Solar PV panel Flow rate indicator (Q_2_)	2-inch diameter pipe Length: 240 m Height difference: 6 m	Intermediate station with 1-cubic-meter buffer tank (1 m height)
Station 3	Pump (P3) Valve (V2) Buffer tank (T_2_) with level indicator (L_2_) Solar PV panel Flow rate indicator (Q_3_)	2-inch diameter pipe Length: 320 m Height difference: 3 m	Second intermediate station with 1-cubic-meter buffer tank (1 m height)
Station 4	Valve (V3) Reserve tank (T_3_) with level indicator (L_3_)	N/A (final destination)	Terminal station with 10-cubic-meter reserve tank (2 m height) Controls inflow to final reservoir Tank maintains water level between 0 m and 1.2 m with inlet at 1.8 m No pump required as water is delivered from Station 3

**Table 2:** Tank and control parameters.

Parameter	Value
$Maximum\;opening\;of\;the\;valve\;(V_{max})$	15 mm
Pipe diameter	2 inches
$Proportional\;gain\;of\;the\;valve\;controller\;(K_{p})\;$	3
Maximum water level $\left(L_{max}\right)$	Buffer tank: 0.8 m Reserve tank: 1.2 m
Minimum water level $\left(L_{min}\right)$	Buffer tank: 0.15 m Reserve tank: 0 m
Initial water level	Buffer tank: 0.2 m Reserve tank: 0 m
Averaging window *(N)*	Power: 2 sequences Flow: 4 sequences
Tank capacity	Buffer tank: 1 cubic meter Reserve tank: 10 cubic meters
Tank height	Buffer tank: 1 m Reserve tank: 2 m
Inlet height	Buffer tank: 0.9 m Reserve tank: 1.8 m
Outlet height	Buffer tank: 0.1 m Reserve tank: -
Power increasing/decreasing step ${(∆}_{p})$	30 Watts
Stepping constant ($k_{s}$)	2
Delay sequence (*D*)	150 sequences
Sampling time (*T_s_*)	5 s
Minimum solar power ${(P}_{min})$	300 Watts

**Table 3:** Specific settings for each scenario.

Scenario	Average Power for Each Station	Additional Events
Scenario 1: Clear sky conditions	1700 Watts	-
Scenario 2: Cloudy sky conditions	1300 Watts	-
Scenario 3: Clear sky with temporary power outage	1700 Watts	15 min power outage
Scenario 4: Variable solar irradiance	1000–2000 Watts	250-Watt increasing step

**Table 4:** System performance correlation.

Average Power (W)	Filling Time to 3 m^3^	Tank Overflow	Tank Shortage	Steady-State Error (%)
1000	37 min	-	-	3.4
1250	34 min	-	-	2.9
1500	33 min	-	-	2.3
1750	32 min	-	-	1.9
2000	31 min	-	-	1.8

**Table 5:** Steady-state power errors at final phase of each scenario.

Test Scenario	Filling Time (min)	Overflow	Shortage	Recovery from Disturbance
Clear Sky	31	No	No	Not required
Cloudy Sky	34	No	No	Not required
Power Outage	35 (incl. outage)	No	No	Full recovery
Variable Solar Irradiance	31–37	No	No	Regulated by controller

## Data Availability

This study did not generate any datasets. All results were derived from simulations based on system modeling and control logic design; therefore, no data are available for sharing.

## Abbreviations

The following abbreviations are used in this manuscript:

DC	Direct Current
MPPT	Maximum Power Point Tracking
PV	Photovoltaic
SCADA	Supervisory Control and Data Acquisition
VSD	Variable-Speed Drive
IoT	Internet of Things
PID	Proportional–Integral–Derivative
LoRa	Long-Range Communication
DC-DC	Direct-Current-to-Direct-Current (Converter)
